# Quadricuspid aortic valve with complete heart block: a double whammy

**DOI:** 10.1186/s43044-024-00572-5

**Published:** 2024-10-29

**Authors:** Mukesh Dhillon, Aditi Sharma

**Affiliations:** 1 Department of Medicine, Command Hospital, Panchkula, India; 2 Department of Pediatrics, Command Hospital, Panchkula, India

**Keywords:** Complete heart block, Congenital heart disease, Quadricuspid aortic valve, Transesophageal echocardiogram, Transthoracic echocardiogram, Infective endocarditis

## Abstract

**Background:**

Developmental abnormalities of aortic valve cusps are relatively common with the bicuspid valve being the most frequently encountered congenital heart disease. However, the quadricuspid aortic valve (QAV) is an exceedingly rare abnormality.

**Case presentation:**

We report a case involving a young, otherwise healthy male who presented with non-exertional syncope and was subsequently diagnosed with complete heart block (CHB). Further evaluation revealed the coexistence of a rare quadricuspid aortic valve and CHB. This combination, in the absence of surgery or infective endocarditis, has only been reported once before in the literature.The patient underwent successful permanent pacemaker implantation and continues to be monitored for aortic regurgitation.

**Conclusions:**

The coexistence of a QAV with CHB, in the absence of infective endocarditis or aortic valve surgery, is extremely rare and necessitates careful evaluation and follow-up.

**Supplementary Information:**

The online version contains supplementary material available at 10.1186/s43044-024-00572-5.

## Background

A Quadricuspid aortic valve (QAV) is an exceedingly rare cardiac anomaly with an estimated incidence of less than 0.05% in the general population [1]. Due to its rarity, the natural history, clinical characteristics, and long-term outcomes of QAV are not well understood, with most cases being small series from single centers [1]. Typically, QAVs are detected when a patient develops hemodynamic abnormalities of the aortic valve. Compared to the tricuspid valve, these valves with abnormal cusps are prone to aortic regurgitation (AR), aortic stenosis (AS), endocarditis, and aortic root dilatation [1–3]. Occasionally, QAVs are identified during the evaluation of associated cardiac structural defects.

Complete heart block (CHB) is a life-threatening condition that can arise as a complication following aortic valve surgery [4]. Only one case of QAV with congenital CHB and another case of QAV with CHB as a complication of infective endocarditis (IE) has been reported in the literature [5, 6]. Therefore, our case represents the second documented instance of a QAV coexisting with CHB without a history of IE or surgical intervention.

## Case presentation

Our patient is a 40-year-old male with no prior known comorbidities and who led an active lifestyle. He presented with an episode of non-exertional syncope, with no associated symptoms such as chest pain, diaphoresis, or seizure-like activity. On arrival at the emergency department, the patient was conscious and alert, with a pulse rate of 45 beats per minute and a blood pressure reading of 146/70mmHg. His respiratory rate and oxygen saturation were within normal limits. On physical examination, normal heart sounds were heard, with the presence of an early diastolic murmur over the aortic area.

**Fig. 1:**
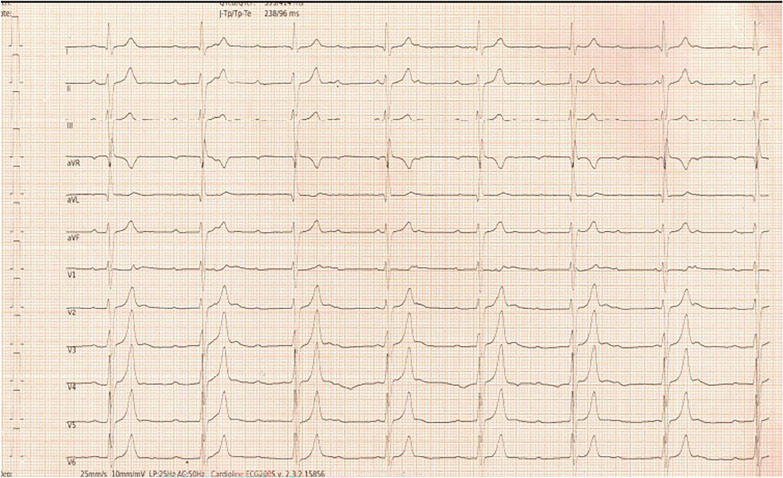
Electrocardiogram (ECG) showing CHB with atrial rate of 115 beats per minute and ventricular rate of 43 beats per minute with narrow QRS

The electrocardiogram (ECG) revealed CHB, characterized by an atrial rate of 115 beats per minute and a ventricular rate of 43 beats per minute with a narrow QRS complex (Fig. 1). Laboratory evaluations including a complete blood count, electrolytes, and thyroid function tests were normal. The patient had no history of autoimmune disease, and an immunological workup, including an antinuclear antibody profile was negative. There was no clinical, radiological, or biochemical evidence of sarcoidosis. The patient also reported no similar medical conditions among family members.

**Fig. 2:**
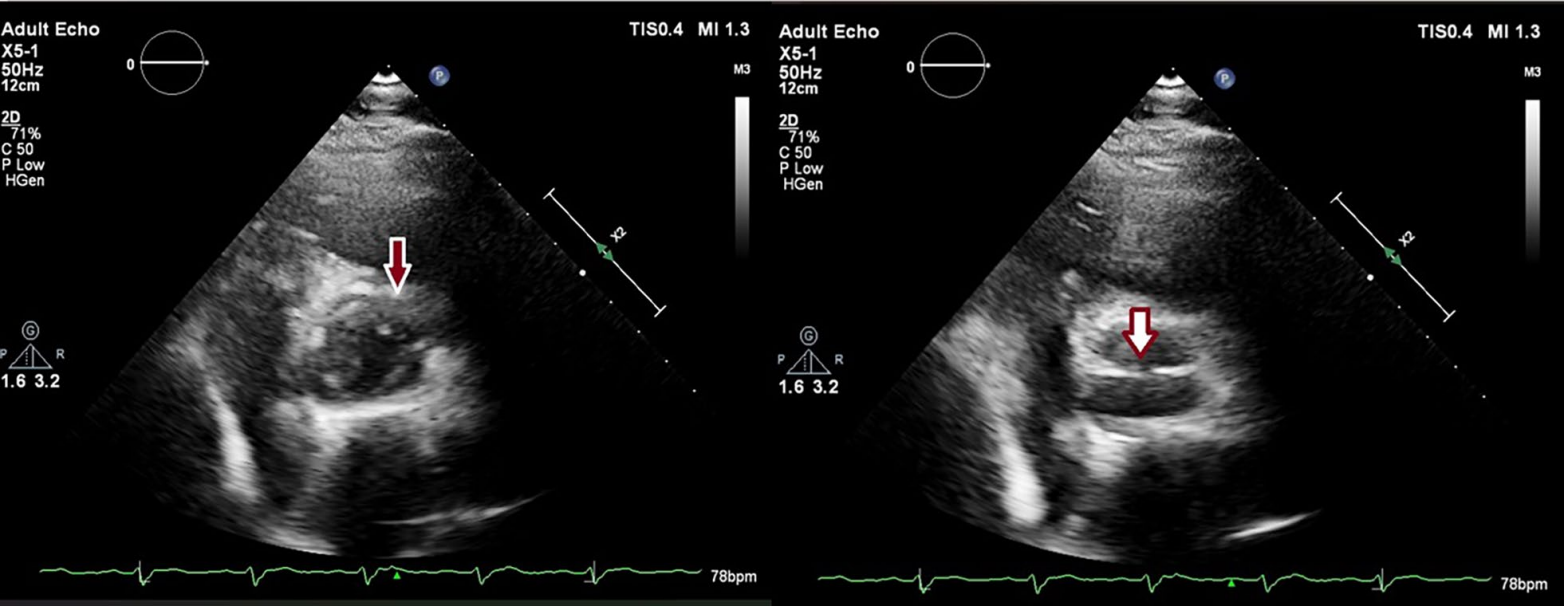
Transthoracic echocardiogram (TTE) image in parasternal short axis showing aortic valve in systole (red solid arrow) and in diastole (white solid arrow). Note the single commissural line in diastole

**Fig. 3:**
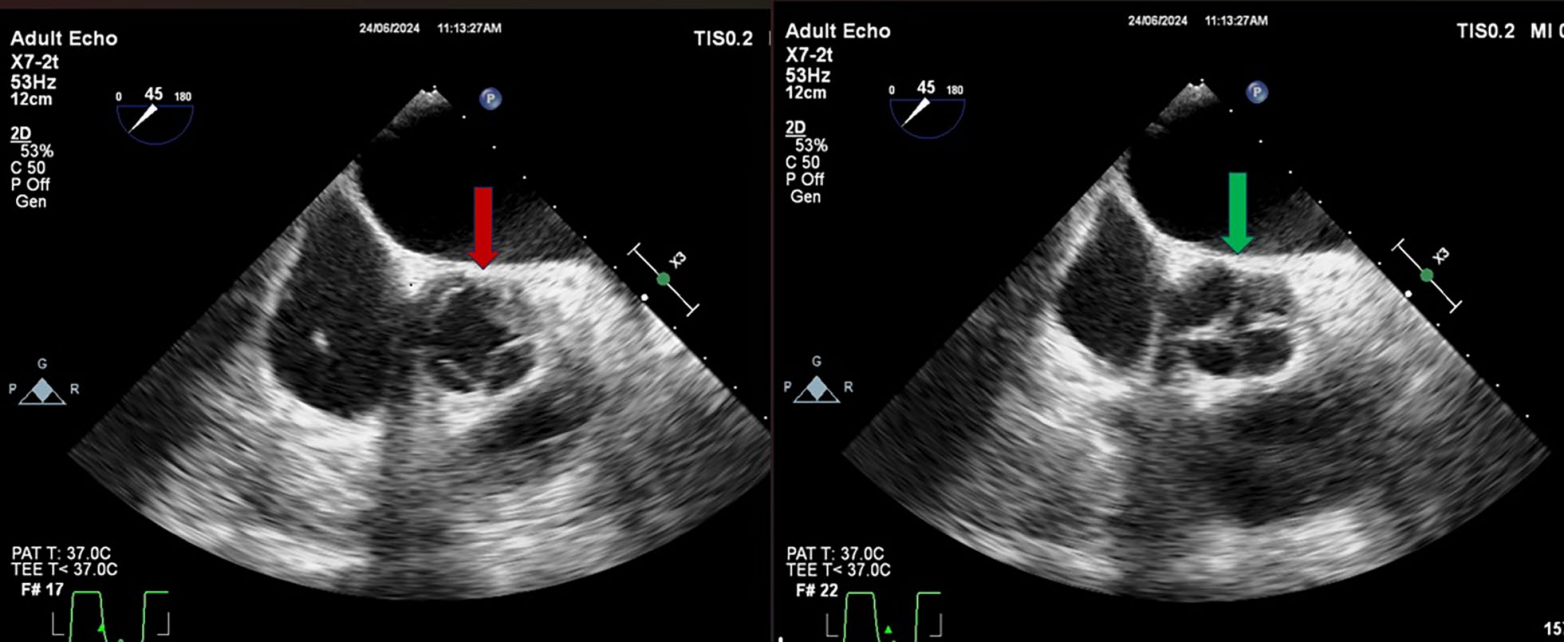
Transesophageal echocardiogram (TEE) image showing aortic valve with 4 cusps in systole (solid red arrow) and diastole (solid green arrow). Note the configuration of “X” in diastole

A transthoracic 2D echocardiogram (TTE) showed normal ventricular function, and normal chamber sizes, with mild eccentric AR. The transvalvular gradients and aortic root size were normal. The aortic valve appeared like a bicuspid with a single commissural line in diastole, however, in systole, it appeared to have 4 cusps (Fig. 2), therefore a transesophageal echocardiogram (TEE) was done which showed it to be quadricuspid (Fig. 3 and supplementary video).

Based on the clinical features, ECG, and echocardiographic results the patient was diagnosed as a case of CHB with underlying QAV. In this clinical scenario, the CHB could be congenital, related to infective endocarditis, or associated with abnormal valve anatomy and hemodynamics. The patient underwent an uneventful dual-chamber permanent pacemaker implantation and was advised to follow up with a repeat TTE every 2 years.

## Discussion

Developmental abnormalities of the aortic valve are frequently observed, with the bicuspid aortic valve being the most common congenital defect, affecting 0.9–1.3% of the population [7]. Other less common variants include unicuspid (0.02%), quadricuspid (0.008%–0.043%), and pentacuspid aortic valves. [7] Advances in echocardiography have facilitated non-invasive diagnosis of these abnormalities, which were previously often detected during surgery or at autopsy. In the short axis view, a QAV typically exhibits an “X” shape in diastole, in contrast to the “Y” pattern of a normal tricuspid valve or a single commissural line characteristic of a bicuspid valve. Our patient's TTE showed a single commissural pattern in diastole; however, on TEE it was confirmed to be QAV (Figs. 2 and 3).

Some pathophysiological mechanisms proposed that might alter the valve cusp number are irregular septation of the conotruncus, anomalous proliferation of mesenchymal ridges, or valve cusp division during its formation. However, as per the most accepted hypothesis, there is an invagination of the endothelial layer on the luminal side causing partition of one of the three-valve cushions during an early stage of valve development [8]. The functionality of a QAV usually remains normal until around 18 years of age, but it tends to deteriorate after the age of 40. There is a slight male predominance in cases of QAV. Clinical manifestations vary and depend on the functional status of the valve, ranging from asymptomatic to palpitations, chest pain, shortness of breath, sudden cardiac death, or features of advanced heart failure [3].

Observational data indicate that AR is more common than AS or ascending aortic valve enlargement in patients with QAV. Unequal cusp size and abnormal shear stress lead to progressive fibrosis and poor leaflet coaptation [8]. Approximately half of all QAV patients will eventually require surgical intervention [2]. Furthermore, one-third of individuals with QAV have other associated structural heart diseases, such as mitral or tricuspid valve prolapse, pulmonary valve stenosis, hypertrophic cardiomyopathy, atrial or ventricular septal defects, or coronary anomalies [1, 3].

Our case is the second reported instance of QAV with CHB in the absence of IE or aortic valve surgery. While CHB may occur due to IE-related abscess formation or infection extending from the aortic valve, these were absent in our patient [6]. Although congenital CHB is a possibility, the patient had never undergone an ECG before his syncopal episode and lived an active lifestyle without symptoms of fatigue. Aortic valve fibrosis and calcification which progresses to involve aortic root is a less studied cause of conduction pathway abnormalities and can be responsible for CHB in our case [9]. The right and non-coronary sinuses of Valsalva lie close to the superior interventricular septum and His bundle, and it may be that hemodynamic stress of spontaneous AR leads to dilatation of aortic annulus and surrounding structures. Unequal size of cusps with uneven distribution of stress was a postulated mechanism for these hemodynamic abnormalities, however, these can occur with equal size cusps as well [3]. Nearly half of patients with QAV will require valve surgery in their lifetime. During the surgical replacement of these valves, the surgical suture should be transitioned to a supra-annular location and anteriorly high within the membranous septum to avoid the development of CHB in the postoperative period [4].

Here we have reported a rare combination of two life-threatening conditions with CHB in a patient with QAV in the absence of IE or surgery. These QAVs are prone to hemodynamic stress leading to AR, AS, and aortic root dilatation. Also, they are at increased risk of IE necessitating surgical replacement. During surgery placement of suture is important to avoid post-operative CHB. After a permanent pacemaker, they need regular follow-ups for the development of hemodynamic abnormalities of AR, AS, and aortopathy due to underlying QAV. It is reasonable to repeat TTE every 2–3 years in patients with mild to moderate AR [10]. It is also important to note that QAV may be missed on TTE, and TEE is more informative.

## Conclusion

We have presented a rare case of a patient with QAV and CHB, unassociated with infective endocarditis or aortic valve surgery. This report emphasizes the importance of using TEE for accurate diagnosis, as QAV may be missed on TTE. We believe that CHB in this case may be attributed to the abnormal cusp anatomy and hemodynamic stress-related changes. When performing surgery on QAV patients, special care must be taken to avoid postoperative CHB. After pacemaker implantation, regular follow-up is crucial to monitor for potential hemodynamic abnormalities, such as AR, AS, or aortopathy associated with the underlying QAV.

## Supplementary Information

Additional file1

## Data Availability

Yes.

## Abbreviations

AR: Aortic regurgitation
AS: Aortic stenosis
AV: Aortic valve
CHB: Complete heart block
ECG: Electrocardiogram
IE: Infective endocarditis
TEE: Transesophageal echocardiogram
TTE: Transthoracic echocardiogram
QAV: Quadricuspid aortic valve
